# Whole-transcriptome analysis delineates the human placenta gene network and its associations with fetal growth

**DOI:** 10.1186/s12864-017-3878-0

**Published:** 2017-07-10

**Authors:** Maya A. Deyssenroth, Shouneng Peng, Ke Hao, Luca Lambertini, Carmen J. Marsit, Jia Chen

**Affiliations:** 1 Department of Environmental Medicine and Public Health, Icahn School of Medicine at Mount Sinai, New York, NY 10029 USA; 2 Department of Genetics and Genomic Sciences, Icahn School of Medicine at Mount Sinai, New York, NY 10029 USA; 3 Department of Obstetrics, Gynecology and Reproductive Science, Icahn School of Medicine at Mount Sinai, New York, NY 10029 USA; 4 Department of Environmental Health, Emory University, Atlanta, GA 30322 USA; 5 Department of Pediatrics, Icahn School of Medicine at Mount Sinai, New York, NY 10029 USA; 6 Department of Medicine, Hematology and Medical Oncology, Icahn School of Medicine at Mount Sinai, New York, NY 10029 USA; 7 Department of Oncological Sciences, Icahn School of Medicine at Mount Sinai, New York, NY 10029 USA

**Keywords:** Placenta, RNA-Seq, WGCNA, Birth weight

## Abstract

**Background:**

The placenta is the principal organ regulating intrauterine growth and development, performing critical functions on behalf of the developing fetus. The delineation of functional networks and pathways driving placental processes has the potential to provide key insight into intrauterine perturbations that result in adverse birth as well as later life health outcomes.

**Results:**

We generated the transcriptome-wide profile of 200 term human placenta using the Illumina HiSeq 2500 platform and characterized the functional placental gene network using weighted gene coexpression network analysis (WGCNA). We identified 17 placental coexpression network modules that were dominated by functional processes including growth, organ development, gas exchange and immune response. Five network modules, enriched for processes including cellular respiration, amino acid transport, hormone signaling, histone modifications and gene expression, were associated with birth weight; hub genes of all five modules (*CREB3*, *DDX3X, DNAJC14*, *GRHL1* and *C21orf91*) were significantly associated with fetal growth restriction, and one hub gene (*CREB3*) was additionally associated with fetal overgrowth.

**Conclusions:**

In this largest RNA-Seq based transcriptome-wide profiling study of human term placenta conducted to date, we delineated a placental gene network with functional relevance to fetal growth using a network-based approach with superior scale reduction capacity. Our study findings not only implicate potential molecular mechanisms underlying fetal growth but also provide a reference placenta gene network to inform future studies investigating placental dysfunction as a route to future disease endpoints.

**Electronic supplementary material:**

The online version of this article (doi:10.1186/s12864-017-3878-0) contains supplementary material, which is available to authorized users.

## Background

It is increasingly appreciated that the intrauterine experience impacts the long-term health of the offspring [1–3]. The placenta is the principal organ regulating the intrauterine environment and orchestrating gestational development. Situated at the maternal-fetal interface, the placenta acts as a multi-organ system, performing at times the functions of the lung, liver, gastrointestinal tract, kidney and endocrine organs on behalf of the developing fetus [4]. Through this capacity, the placenta plays an active role in facilitating the transport of nutrients and waste, exchange of gases, immune protection and generation of hormones to carefully regulate fetal growth and organ-specific development throughout gestation [4]. Alterations to the intrauterine environment through the action of various intrinsic and extrinsic stressors can impact placental function, including vascularization, growth, transport activity, metabolism and hormone production, and as a result, impact the physiology of the developing fetus, with potential health consequences across the lifespan [5, 6]. The placenta, therefore, is the primary regulator through which the developmental trajectory of the fetus is altered in response to changes in the intrauterine environment [7]. Despite the centrality of the placenta in the developmental origins of health and disease, this vital organ remains poorly characterized.

Recent developments in high throughput whole-genome sequencing make it feasible to assess the entire transcriptome with the potential to agnostically uncover biological processes driving complex phenotypes. Studies assessing the transcriptome-wide profile of human term placenta are beginning to emerge [8–11], however, the inferences from these findings are limited by the small sample size and the focus on univariate gene expression analyses contrasting normal and adverse phenotypic outcomes. Systems biology methods that apply a networks-based analysis of transcriptome-wide data better capture the complexity of inter-gene relationships and the pathways they participate in to give rise to disease processes, and, therefore, hold the opportunity to better define the co-regulatory patterns that underlie complex phenotypes. Methods such as weighted gene co-expression network analysis (WGCNA) have been successfully applied in a number of tissues in relation to various health outcomes [12–15]. This approach facilitates systems-level characterization of expression changes by clustering highly correlated genes into coexpression modules of conserved biological function [16, 17]. The utility of this approach was demonstrated in a recently published study focusing on highly variable genes (n ~ 3000) in a set of 16 placenta that showcased both conservation and divergence between human and mouse placental networks [18].

Abnormal fetal growth, both undergrowth (fetal growth restriction) and overgrowth (macrosomia), is among the most commonly reported outcomes linked to placental dysfunction. Infants clinically defined as either small (SGA, bottom 10% weight for gestational age) or large (LGA, above 90% weight for gestational age) are of particular concern as they are at elevated risk for postnatal morbidities, including metabolic syndrome and impaired neurobehavioral and cognitive development [19–24], highlighting the public health impetus to identify the molecular mechanisms underlying fetal growth dysregulation. Hence, this outcome provides an exemplary opportunity to demonstrate the importance of placenta gene networks by elucidating fetal-growth related placental processes. In addition, these findings have the potential to be translated into novel approaches for disease intervention or even prevention at an early time-point, when they may be the most effective.

In this study, we comprehensively profiled the transcriptome-wide landscape of the largest birth cohort collection of human placentae to date, implementing a network-based approach to construct a placental gene coexpression network and to delineate a fetal-growth gene signature.

## Results

The demographic characteristics of our study population, categorized as small (SGA), large (LGA) and appropriate (AGA) for gestational age infants, are shown in Table 1. Consistent with the literature, a greater proportion of LGA infants were male [25] and born to mothers with elevated pre-pregnancy BMI [26]. A greater proportion of SGA infants were female and born to mothers of non-Caucasian descent [27]. Additionally, LGA infants were more likely to be delivered by cesarean section.

**Table 1:** Demographic characteristics of the study population (*n* = 200)

Variables	SGA^a^ (*n* = 33)	AGA^b^ (*n* = 112)	LGA^c^ (*n* = 55)	*p*-value^d^
	Mean (SD)	Mean (SD)	Mean (SD)	
Birth weight (g)	2582.3 (277.3)	3436.8 (388.9)	4276.9 (247.2)	<0.01
Gestational age (weeks)	38.9 (1.2)	39.1 (0.9)	39.0 (0.8)	0.77
Maternal age (years)	31.9 (5.6)	31.1 (4.6)	30.9 (4.1)	0.59
Maternal BMI (kg/m^2^)	25.7 (7.0)	25.6 (5.8)	28.4 (6.9)	0.02
	N (%)	N (%)	N (%)	
Infant Gender				0.05
Female	22 (66.7)	57 (50.9)	22 (40.0)	
Male	11 (33.3)	55 (49.1)	33 (60.0)	
Maternal Ethnicity				<0.01
Caucasian	18 (54.5)	91 (81.2)	46 (83.6)	
African American	6 (18.2)	2 (1.8)	3 (5.5)	
Other	7 (21.2)	19 (17.0)	5 (9.1)	
Unknown	2 (6.0)	0 (0.0)	1 (1.8)	
Delivery Method				
Vaginal	19 (57.6)	65 (58.0)	19 (34.5)	0.01
C-Section	14 (42.4)	47 (42.0)	36 (65.5)	

^a^small for gestational age; ^b^appropriate for gestational age; ^c^large for gestational age; ^d^ *p*-values based on chi-square test (categorical variables) or ANOVA (continuous variables)

The RNA-Seq dataset averaged 44 million reads per sample. Of all annotated NCBI Reference Sequence genes (*n* = 23,228), 22,518 genes were mapped at least one count per sample, with 52% genes surpassing expression levels above 2 in the log2 scale in a minimum of 30 samples. Among 20 samples run in triplicate, we observed a median R^2^ value of 0.97, with a range between 0.93 and 0.98. Consistent with previous reports [8, 9], the most abundant placental transcripts observed in our study include placental hormones (*CSH1* [28]), placental development factors (*PAPPA* [29], *PAPPA2* [30]), steroidogenesis enzymes (*CYP19A1* [31]), tissue remodeling genes (*TFPI2* [32]*, FBLN1* [33]*, FN1* [11, 34]*, MALAT1* [35]*)*, ribonucleases (*RPPH1* [36]) and signaling molecules (*RHOBTB3)* [37]. Several of the most abundant transcripts are putative imprinted genes, including *TFPI2* [38], *RHOBTB3* and *PAPPA2* [37] (Additional file 1)*.* For technical validation of the RNAseq results, we additionally examined the agreement between RNASeq and the probe-hybridization based nCounter (NanoString technologies, Seattle, Washington) method for a subset of genes with expression data available on both platforms (*n* = 80) [39]. A significant, positive correlation was observed in gene rankings based on relative gene expression levels (Spearman rho = 0.76), indicating relative gene expression levels were comparable across the platforms (Additional file 2).

### Placental gene coexpression network

**Fig. 1:**
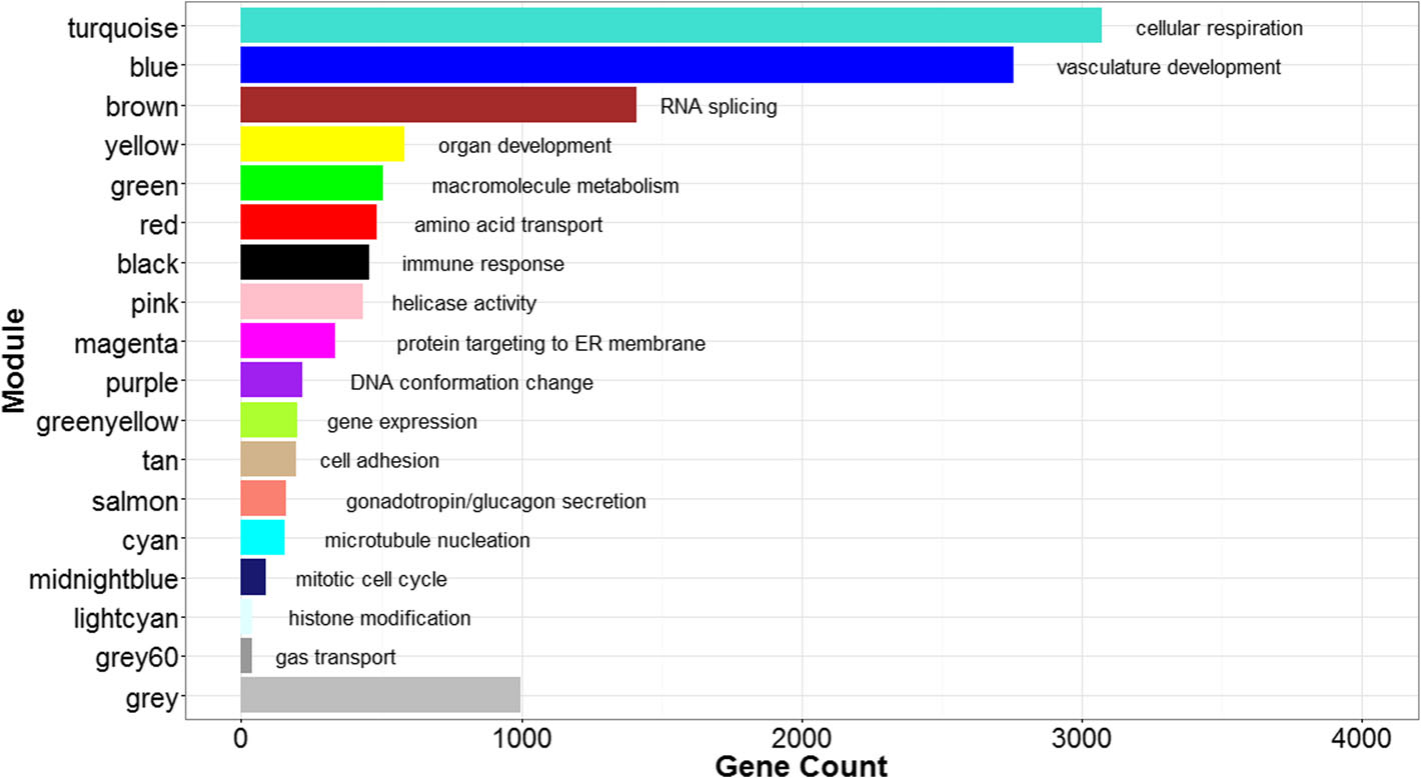
Placental gene coexpression network. We identified 18 network modules ranging in size from 37 to 3073 genes. A total of 998 genes (*grey module*) did not load onto any specified module. Gene ontology enrichment analysis revealed key growth and developmental processes enriched in each module, including transcriptional activity, cell division and respiration

**Fig. 2:**
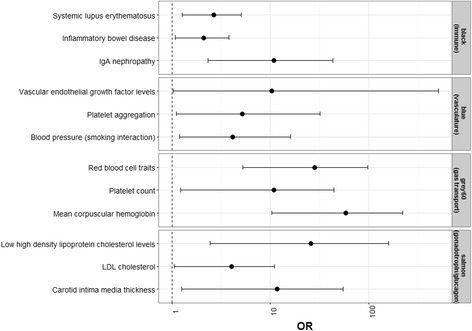
Enrichment of GWAS-associated phenotypes in network modules. Enrichment odds ratios (OR) and 95% confidence intervals are shown for a subset of phenotypes observed to be significantly enriched in the *black*, *blue*, *grey60* and *salmon modules*

Network-based analyses provide a means to account for the coordinated expression among genes, thereby reducing the dimensionality of the data-set and affording insight into underlying biological processes. Coexpression network analysis of the placental transcriptome revealed 17 coexpressed gene modules in human placenta (Additional file 3). Module size ranged from 37 to 3073 genes (Fig. 1). Approximately 1000 genes did not load onto any specific module (*grey* module). To identify biological functions associated with each module, we performed gene ontology (GO) analysis. While there were overlapping processes enriched across several modules, including processes related to chromatin assembly (*purple* and *light cyan*), most processes were uniquely enriched in specific modules, including immune response (*black* module), gas transport (*grey60*) and cell adhesion (*tan*). Modules enriched in similar biological processes tended to cluster together using unsupervised hierarchical clustering (Additional file 4). For example, both *brown* and *greenyellow* modules are involved in mRNA processing and *black* and *tan* modules are involved in extracellular signaling. Next, we investigated whether disease signatures are present within the placental network. For this purpose, we surveyed a curated list of published GWAS, including 23,086 genes and spanning 760 phenotypes, for the enrichment of genes with phenotype-linked variants within our placental network. Overall, 651 GWAS-linked genes encompassing 185 phenotypes were enriched in the placental network (*p* < 0.05) (Additional file 5). We observed notable trends consistent with the enriched GO-derived biological processes identified in our network modules. For instance, the immune response module (*black*) was enriched for immune-related disorders, such as systemic lupus erythematosus, inflammatory bowel disease and IgA nephopathy; the vasculature development module (*blue*) was enriched in vascular endothelial growth factor levels, platelet aggregation and blood pressure; the gas transport module (*grey60*) was enriched in red blood cell traits, platelet count and mean corpuscular hemoglobin; and the module involved in the secretion of the metabolic hormones gonadotropin/glucagon (*salmon*) was enriched in low high density lipoprotein cholesterol levels, LDL cholesterol and carotid intima media thickness (Fig. 2).

We defined the 1st principal component of each module, the module eigengene, as a summary measure of each module [17, 40]. Genes most strongly correlated with the eigengene of each module (hub genes) are shown in Table 2. In most instances, the identified hub genes reflect the biological processes enriched in the modules. For example, complement system genes are the hub genes of the immune response module (*black*), ribosome-coding genes are the hub genes of the protein translation module (*magenta*) and histone modification genes are the hub genes of the DNA conformation change module (*purple*).

**Table 2:** Placenta gene coexpression network hub genes

Module	Hub gene
Turquoise	*HELZ*, *MLL2*, *YIF1*, *C12orf5*, *GHITM*
Blue	*TCF4*, *PDGFRB*, *ETS1*, *COL6A2*, *SPTBN1*
Brown	*RBM33*, *ATXN2L*, *MYSM1*, *MLL4*, *CBFA2T2*
Yellow	*ARHGAP23*, *LAMB1*, *UTRN*, *LRP5*, *MYH10*
Green	*UBE2W*, *TMED7*, *ZNF24*, *SOCS6*, *RAP2C*
Red	*FAR1*, *B4GALT3*, *POR*, *PIGH*, *NISCH*
Black	*C1QC*, *C1QB*, *CD68*, *CTSS*, *C1QA*
Pink	*MPZL1*, *SKP2*, *MCM3*, *BMP7*, *MCM*
Magenta	*RPL7A*, *UBA52*, *RPS5*, *RPL18*, *RPS11*
Purple	*HIST1H3F*, *HIST1H2AH*, *CDK1*, *FEN1*, *CDC20*
Greenyellow	*C21orf91*, *ZNF721*, *INO80D*, *ANKRD12*, *MBTD1*
Tan	*NOTUM*, *HN1*, *REPS2*, *B3GNT7*, *PYCR1*
Salmon	*PVRL4*, *NDRG1*, *GRHL1*, *PLIN2*, *HMHA1*
Cyan	*ZC3H18*, *UBE2N*, *PRDX3*, *CAPZA2*, *CDK12*
Midnight blue	*TOP2A*, *TPX2*, *LMNB1*, *CENPF*, *SLC13A3*
Light cyan	*DDX3Y*, *RPS4Y1*, *KDM5D*, *ZFY*, *TTTY15*
Grey60	*ALAS2*, *SLC4A1*, *HBA1*, *HBG1*, *HBA2*

**Fig. 3:**
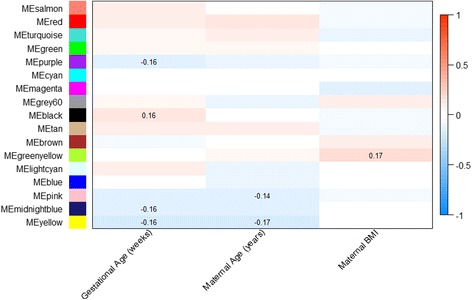
Spearman correlations between placenta network modules and continuous demographic characteristics. The color gradient indicates the direction, positive (*red*) and negative (*blue*), and strength of the correlation. Significant correlation coefficients (*p* < 0.05) are indicated on the plot

**Fig. 4:**
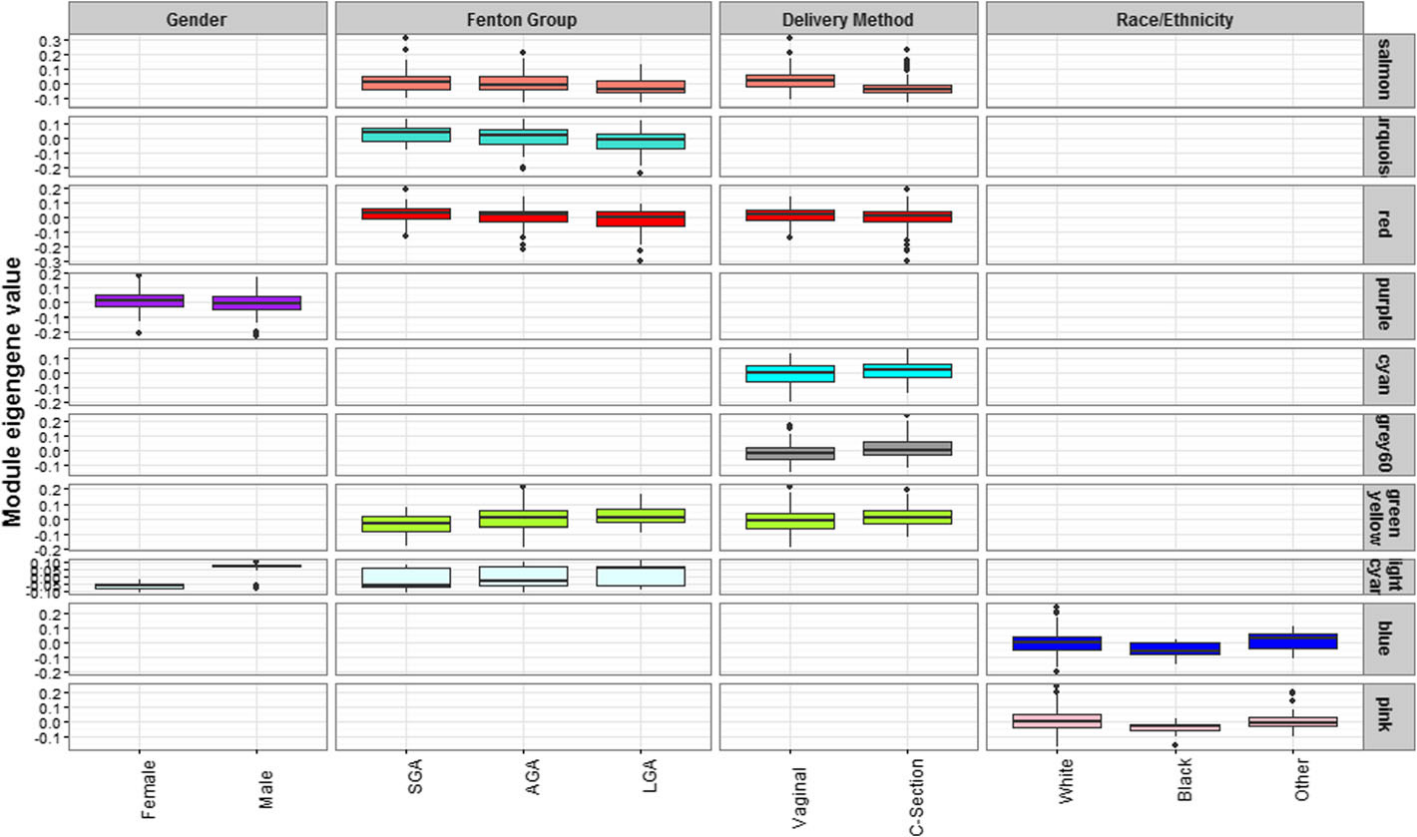
Placenta network module differences across categorical demographic characteristics. Significant differences were determined based on Mann Whitney U tests (2 groups) and Kruskal Wallis tests (>2 groups)

Correlations between network module eigengenes and continuous demographic variables are shown in Fig. 3. Several interesting patterns are apparent. With increasing gestational age, a negative correlation with drivers of growth and development (*purple*, *midnightblue* and *yellow*) and a positive correlation with immune-related activity (*black*) was observed. An increase in maternal age was negatively correlated with processes related to development (*yellow*) and cell replication (*pink* and *midnight blue*), while an increase in maternal BMI was correlated with an upregulation of growth-promoting processes (*greenyellow*). Differences across categorical demographic variables were evaluated based on mean differences in module eigengene values. As seen on Fig. 4, module eigengene values were significantly different with respect to infant gender, maternal race/ethnicity, delivery method and birth weight categories. Differences across infant gender were observed in modules involved in chromatin assembly-related processes (*light cyan* and *purple*). An upregulation of growth-promoting processes was observed among white infants compared to non-white infants (*blue* and *pink*). Differences in modules involved in growth and development related processes, including respiration (*turquoise*), amino acid transport (*red*), metabolic hormone secretion (*salmon*), gene expression (greenyellow) and histone modification (*light cyan*), were observed across birth weight categories. Several modules related to birth weight overlapped with modules related to gender (*light cyan*), maternal BMI (*greenyellow*) and delivery method (*salmon*, *red*, *greenyellow*).

### Placental gene network modules associated with birth weight

**Fig. 5:**
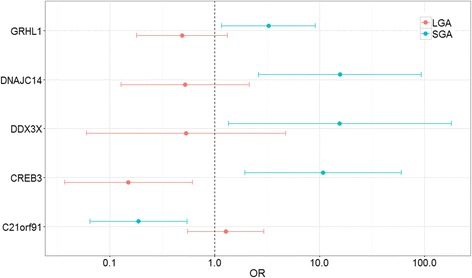
Association between candidate module hub genes and birth weight. Genes shown along y-axis are putative fetal growth-related intramodular hub genes: *CREB3* (*turquoise*), *DNAJC14* (*red*), *GRHL1* (*salmon*), *DDX3X* (*light cyan*) and *C21orf91* (*greenyellow*). Each gene’s association with aberrant fetal growth categories is indicated by the odds ratios (OR) and 95% Confidence Intervals (CI) of SGA and LGA infants referenced against AGA infants for a log2 unit increase in expression. Multinomial regression models were adjusted for infant gender and maternal BMI

To demonstrate the functional relevance of the placenta network identified above, we further evaluated module-based differences in fetal-growth related gene pathways. Intramodular hub genes are genes with maximal connections to other genes in their respective modules. Given the importance of these genes in likely determining the behavior of the module-associated biological pathway, we identified the candidate fetal growth-related intramodular hub genes within each of the five fetal growth-related modules, focusing on genes strongly correlated with both Fenton growth curve percentiles and module eigengene values (Additional file 6). Significant associations with deviations in appropriate fetal growth are observed among genes with high module membership (Fig. 5), including *CREB3* (*turquoise* module), *DNAJC14* (*red* module), *GRHL1* (*salmon* module) and *C21orf91* (*greenyellow* module). A log2 unit increase in the expression of *CREB3*, *DNAJC14, DDX3X* and *GRHL1* is associated with increased odds of SGA status, while a log2 unit increase in the expression of *C21orf91* is associated with decreased odds of SGA status. A log2 unit increase in the expression of *CREB3* is additionally observed to be protective against LGA status. Comparing the candidate fetal growth-related intramodular hub genes with the overall module hub genes listed in in Table 2, an overlap of two *salmon* module genes, *GRHL1* and *PVRL4* as well as one *greenyellow* module gene, *C21orf91*, was observed.

**Fig. 6:**
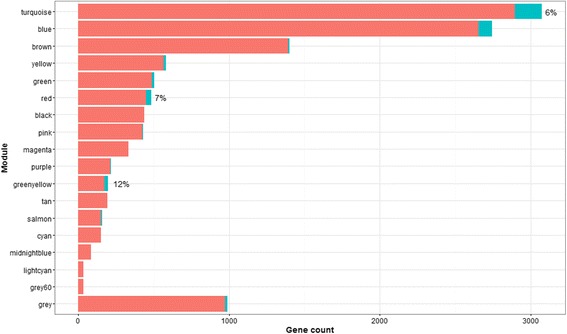
Enrichment of genes differentially expressed by birth weight in network modules. Twelve modules contained genes differentially expressed by birth weight category (*n* = 393). The proportion of differentially expressed genes within each of the modules is indicated in *blue* in the stacked bar plot. A significant enrichment of differentially expressed genes based on a fisher’s exact test was observed in the *turquoise*, *red* and *greenyellow* modules

Conventional univariate analyses across all 12,095 genes indicated 393 genes differentially expressed across the three birth weight categories, primarily between SGA and LGA infants. Genes differentially expressed across the birth weight categories predominately loaded onto modules differentially coexpressed across birth weight categories, with a significant enrichment observed in the *turquoise*, *red* and *greenyellow* modules (Fig. 6).

**Fig. 7:**
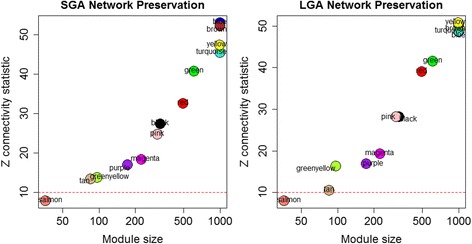
Loss of network conservation in SGA and LGA networks compared to AGA networks. Relative to the AGA network, the salmon module demonstrates loss of connectivity in the SGA (Z = 7.85) and LGA (7.95) networks

In addition to the global network, we also constructed separate networks for each birth weight category. Fewer modules are observed in the separate networks compared to the global networks, with 15 modules in the SGA network, 12 modules in the LGA network and 13 modules in the AGA network. Next, we compared the network density and connectivity patterns of the SGA and LGA networks to the AGA network to determine whether the topology of the AGA network is preserved in the aberrant fetal growth networks. Network density measures assess whether gene-sets that load onto a common module in the reference network are also adjacent to one another in the test networks, while network connectivity measures assess whether the connection strengths of module gene-pairs in the reference network are preserved among the same gene-pairs in the test networks. While no differences in network densitity measures were observed, as shown in Fig. 7, a significant loss of network connectivity (Z statistic <10) was observed in the salmon module for both the SGA (Z connectivity =7.85) and LGA (Z connectivity = 7.95) networks compared to the AGA network. This loss in network connectivity suggests that a subset of gene interactions in the salmon module are disrupted in the aberrant fetal growth categories, indicative of a breakdown in the associated biological pathway.

**Fig. 8:**
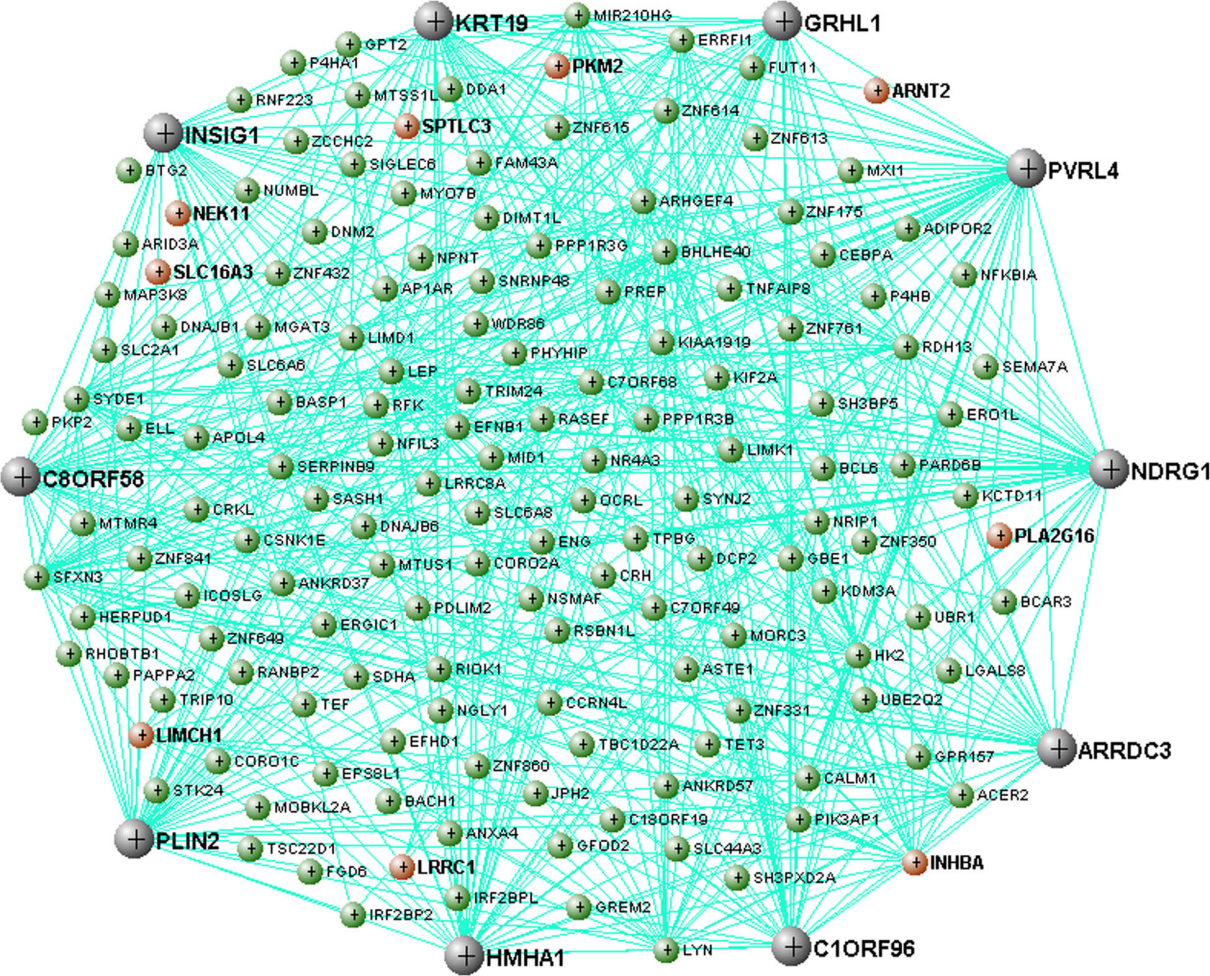
Gene connectivity in the *salmon* module. *Black* nodes indicate the most highly connected genes in the module (hub genes). *Red* nodes indicate genes differentially expressed across birth weight categories

An example of module connectivity patterns, indicating both genes differentially expressed across birth weight categories and module hub genes, is shown for the *salmon* module in Fig. 8. With the exception of *INHBA*, the differentially expressed genes largely demonstrate weaker connectivity patterns within the module in relation to the hub genes.

## Discussion

In this study, we have taken a systems biology approach to describe the functional gene networks present in the human term placenta. Weighted gene coexpression analysis of the placental transcriptome revealed an enrichment in functional processes related to growth and development, including cellular respiration, transcriptional activity, and signal transduction. Although few descriptions of non-pathological tissue-specific coexpression networks are available in the literature, a few general trends are observable. Placental modules enriched for biological processes involved in transcriptional activity, cellular respiration and immune response were also common to processes observed in other reported tissue networks, including liver [41], skeletal muscle [42] and blood [43]. Compared to these networks, processes uniquely enriched in the placenta include organ and vasculature development, cell replication, cell adhesion, gas transport and hormone secretion. Furthermore, the processes enriched in the placental gene network reflect known critical functions perfomed by the placenta for appropriate fetal development, including establishing a blood supply (vasculature development), trophoblast adherence to maternal decidua (cell adhesion), gas exchange (gas transport), feto-maternal immune tolerance (immune response) and endocrine signaling (hormone secretion). Interestingly, genes with common GWAS-associated biomedical traits also loaded onto our network modules. While the molecular function/process assigned to genes using GO are based on curated in vitro or *in silico* evidence of participation in biological pathways [44], the GWAS catalog comprises array-based genotyping studies conducted in human population settings [45, 46]. Hence, our observed GWAS-enrichments corroborate the functional processes assigned to the modules based on GO enrichment analyses and also suggest the possible priming of later life health effects during development.

Several network modules were observed to be related to maternal-infant demographic variables, suggesting that the activity of these modules reflect gestational characteristics. For example, the observed downregulation in growth-related processes with increasing gestational age may indicate a shift in placental processes to ready the fetus for postnatal life. Similarly the gestational age associated upregulation of immune-related processes is consistent with the onset of parturition [47, 48]. Given the narrow range in gestational age in our study population, however, our observations, while consistent with known trajectories in feto-placental development, would require further in-depth physiologic analysis to establish biologic relevance. Several modules were associated with multiple demographic characteristics. For example, an upregulation of the histone modification (lightcyan) module was observed both among male infants and LGA infants, consistent with known trends in gender differences based on birth size. The upregulation of the gene expression (greenyellow) module with increasing maternal BMI and birth weight is also consistent with a posititve relationship between maternal BMI and birth size. Similarly, changes in modules by delivery status tracked closely with patterns of modules distinguishing LGA and AGA infants, consistent with observations in our study that LGA infants were more likely to be delivered by cesarean section.

To evaluate the utility of the derived placental gene network in elucidating the molecular underpinnings of placental dysfunction, we examined fetal growth-related perturbations to the network. The network-based analysis reduced the dimensionality of the data and accounted for the interdependent structure among the transcripts, enabling us to derive functional insight into the biological processes affected by fetal growth-related dysregulation of coexpression. In our study, we identified 5 network modules with differential coexpression across birth weight categories that are functionally enriched for cellular respiration (*turquoise*), amino acid transport (*red*), gonadotropin/glucagon secretion (*salmon*), gene expression (*greenyellow*) and histone modification (*light cyan*). Several of these modules, ones related to cellular respiration, amino acid transport and gene expression, were also significantly enriched with genes differentially expressed across birth weight categories.

A loss in overall network connectivity in SGA and LGA infants was observed for the gonadotropin/glucagon secretion (*salmon*) module, highlighting a breakdown in key gene interactions within this module among the aberrant growth categories. A closer look at the salmon module highlights the added value gained by using a networks-based approach. This module includes differentially expressed genes previously shown to be relevant to fetal growth and development, i.e., *PKM2* [49], *SPTLC3* [37] and *INHBA* [50]. Additionally, this module includes genes that were not identified as differentially expressed in the current study but have been previously shown to be relevant to fetal growth and pregnancy complications in independent studies, primarily *LEP* [51], as well as the hub genes *PVRL4* and *PLIN2* [52]. The fact that these genes, which were previously independently implicated with fetal growth and development, load onto a common module in the current study suggests that the mechanism through which these genes exert their effect may converge on a common biological pathway.

While the genes we observed to be differentially expressed were enriched in modules where we observed differential coexpression, it is of interest to note that none of the differentially expressed genes represent hub genes of fetal growth related modules. In fact, differentially expressed genes tended to be relatively weakly connected within network modules. This highlights a key distinction between differential expression and network-based coexpression analyses. Coexpression networks follow a small world architecture, whereby the presence of highly connected hub genes facilitates information flow through the network in relatively few hops [53]. Due to this small world structure of coexpression networks, there is redundancy in the network, allowing for tolerance in perturbations among the majority of nodes. Large-scale perturbations among the handful of highly connected nodes, however, would result in network failure [53, 54]. As differential expression analysis identifies genes with the largest fold changes, these genes are likely situated at the periphery of the network, whereas genes central to fetal growth-related biological processes may be missed. Additionally, the fact that the majority of genes in modules observed to be relevant to fetal growth were not observed to be differentially expressed also highlights that disrupted coexpression in a module can occur in the absence of net differential expression among the individual genes within the module. This suggests that network-based methods may be more robust at uncovering key determinants of affected biological processes and potentially more relevant interventional targets than differential expression analysis.

Several limitations in our study design warrant further discussion. The placenta is a complex organ, consisting of various cell sub-types with transcriptional profiles that likely vary. Nevertheless, our placental samples were collected from four quadrants exclusively on the fetal membrane side and within 2 cm from the cord insertion site, a region identified as the least variable in the placenta [55]. Furthermore, as our study represents a single assessment at term, a causal link between gene expression and birth weight cannot be drawn. As such, follow up studies are warranted to determine whether placental gene signatures bear relevance on postnatal health, extending into childhood as well as adulthood. Such studies also have the potential to additionally provide further mechanistic insight into the roots and consequences of abnormal fetal growth.

## Conclusions

The current study is the largest comprehensive human placental profiling study to date. The generated profile stems from non-pathological, term placenta, providing a characterization of the baseline placental transcriptional landscape and the role that normal variation might play in defining patterns of intrauterine development in the general population. Importantly, we implemented a systems biology-based approach to characterize the placental gene networks, and we demonstrated the utility of this approach by delineating a fetal growth-related placental gene signature. This signature highlights the potential for leveraging the generated placental gene network to uncover novel insight into the molecular underpinnings of placental dysfunction. This includes the identification of both established and novel genes related to fetal growth, and importantly, the interrelationship among these genes in common deregulated biological pathways. As such, these findings highlight the placenta, a temporal organ that is commonly discarded, as a valuable resource for identifying biomarkers relevant to fetal development and postnatal health effects.

## Methods

### Placenta collection

Placenta tissues were collected as part of the Rhode Island Child Health Study (RICHS), a birth cohort representing the populations of Rhode Island and Southeastern Massachusetts, USA [56]. This population consists of singleton, term infants (≥37 weeks gestation) born without congenital or chromosomal abnormalities and born to women without life-threatening pregnancy complications. The cohort includes large for gestational age (LGA, >90% 2013 Fenton Growth Curve), small for gestational age (SGA, <10% 2013 Fenton Growth Curve) and adequate for gestational age (AGA) infants. Given an a priori interest to study fetal growth, this population was oversampled for both LGA and SGA infants. Of the entire RICHS cohort (*n* = 841), a subset of 200 subjects, representative of the full cohort (Table 1), were selected for RNA sequencing and subsequent analyses. All subjects provided written informed consent approved by the Institutional Review Boards at Women and Infants Hospital and Emory University.

Placental biopsies free of maternal decidua were excised from four quadrants within 2 cm of the cord insertion site, placed in RNALater at 4 °C within 2 h of delivery and at least 72 h later were removed from RNALater, pooled, snap-frozen, homogenized to powder, and stored at −80 °C.

### RNA sequencing

Total RNA was isolated from homogenized placental tissue using the RNeasy Mini Kit (Qiagen, Valencia, CA) and stored in RNAse-free water at −80 °C. The yield was quantified using a Quibit Fluorometer (Thermo Scientific, Waltham, MA) and the integrity was assessed using an Agilent Bioanalyzer (Agilent, Santa Clara, CA). Ribosomal RNA was removed using a Ribo-Zero Kit [57]. RNA was converted to cDNA using random hexamers (Thermo Scientific, Waltham, MA). Transcriptome-wide 50 bp single-end RNA sequencing was conducted using the HiSeq 2500 platform (Illumina, San Diego, CA) [58]. Samples were run in three sequencing batches, with 10% of the samples run in triplicate within each batch.

### Statistical analysis

#### QC filtering and normalization

The raw RNA sequencing data (fastq files) were assessed for quality control, including read length and GC content, using the FastQC software. Reads that passed the quality control metrics were mapped to the human reference genome (hg19) in a splice-aware manner using the Spliced Transcripts Alignment to a Reference (STAR) aligner [59], with common SNPs in the reference genome masked prior to alignment. Genes with counts per million <1 in greater than 30 samples (the sample size of the smallest phenotypic group in this study) were considered unexpressed and removed. Read counts were adjusted for GC content using the EDASeq R package [60], followed by TMM correction for library size differences across samples using the calcNormFactors function in edgeR R package [61]. The data was then transformed into logCPM values accounting for the mean-variance relationship in the data using the voom function of the limma R package [62]. Following assessements of Pearson correlations in gene expression among the triplicate samples, duplicated repeat samples were removed from the analysis. Finally, the normalized log2 counts per million (logCPM) reads were filtered to genes with expression levels above 2 in the log2 scale in a minimum of 30 samples. The final filtered, normalized data-set included 12,135 genes. Eight percent of the variability in our data-set was attributable to batch as determined by the implementation of the pvca R package [63, 64]. Expression level differences based on demographic characteristics were assessed based on Mann Whitney U test for variables with two categories, Kruskal-Wallis test for variables with >2 categories, and Spearman correlations for continuous variables.

#### Weighted gene coexpression network

Prior to constructing the co-expression network, the gene expression data was adjusted to remove potential confounding due to batch effect using the ComBat function in the R package sva [65]. The gene co-expression network was generated using the WCGNA R package [17]. Briefly, a similarity matrix was generated from the normalized RNA-Seq data using absolute values of Pearson correlation coefficients among all gene pairs {s_ij_ = |cor(x_i_,x_j_)|}. The similarity matrix was transformed into an adjacency matrix using an adjacency function based on a weighted soft threshold (β =6) {a_ij_ = s_ij_^β^}. The selected β parameter value satisfied the minimum value required to generate a scale-free topology network (linear regression R^2^ ≥ 0.8). The components of the resulting adjacency matrix indicate connection strengths among gene pairs, with connections among strongly correlated genes emphasized and weakly correlated genes suppressed.

To delineate modules, the relative interconnectedness between each node pair was calculated as a topological overlap similarity measure. Here, the topological overlap for each node pair was defined as the proportion of node connections that are shared between the two nodes out of the total number of node connections of the node with fewer connections, thereby, capturing the similarity in the coexpression relationship with all other genes in the network. The reciprocal topological dissimilarity matrix was used as input for hierarchical clustering, and gene modules were defined based on hierarchical clustering guided by topological overlap, using a dynamic tree cut algorithm to establish modules [66]. Finally, highly correlated modules were merged based on a merging threshold set at a height cut-off of 0.25. In the resulting network, as neighbors in a cluster share high topological overlap, the resulting modules likely indicate a common functional class. As a summary measure of the gene modules, each module was defined by the first principal component of each module (module eigengene) to represent the weighted average gene expression profile of the module. Module membership of genes within each module was determined based on the correlation between individual gene expression values and the module eigengene value. Top five genes based on module membership (correlation with module eigengene) were classified as hub genes. Network modules were further assessed for enrichment of biological processes across Gene Ontology (GO) categories based on Fisher’s exact test. Similarly, Fisher’s exact test was applied to assess network module enrichment of genes linked to specific phenotypes (diseases/traits) based on a curated catalog of genome wide association studies (GWAS) [46, 67]. Phenotypes included in the analysis were restricted to those associated with a minimum of 10 reported gene variants. Fisher’s exact test was applied to trait/module gene membership contingency tables with non-zero cell counts. Spearman correlations were calculated between module eigengenes and continuous mother-infant demographic and gestational variables. Significant differences (*p* < 0.05) in module eigengene values across categorical mother-infant demographic and gestational variables were determined using a Mann-Whitney U test (2 categories) or a Kruskal Wallis test (>2 categories).

#### Network-based differential gene coexpression analysis

Module hub genes related to fetal growth were identified as genes highly correlated with the Fenton growth curve percentile r > |0.2| and the module eigengene (r > |0.8|). To assess the association between the expression of these candidate hub genes and fetal growth, we conducted multinomial regression models using the nnet R package [68], setting SGA and LGA status as the outcomes referenced against AGA status. Models were adjusted for infant gender, delivery method and maternal prepregnancy body mass index (BMI). These variables were selected based on observed differences in eigengene values of modules related to fetal growth (infant gender and *lightcyan* module; delivery method and *salmon*, *red* and *greenyellow* modules; maternal prepregnancy BMI and *greenyellow* module).

To assess whether module topology patterns are altered among the adverse growth phenotypes, separate networks were generated for each birth weight category as described for the global network. Module conservation between the reference AGA network and the SGA and LGA networks was evaluated based on four network connectivity and four network density measurements. Network density measurements assess whether genes that are highly connected in the reference network (assigned to a common module) are also highly connected in test networks. Density preservation was assessed based on the correlations between module genes in the reference network and the corresponding genes in the test network on measures of mean correlation, mean adjacency, mean module membership, and proportion of variance explained by the module eigengene. Network connectivity measurements assess whether connection strengths among module gene-pairs are conserved in reference and test networks. Connectivity preservation was assessed based on the correlations between module genes in the reference network and the corresponding genes in the test network on measures of intramodular connectivity (conservation of hubs; rowsum of adjacency), adjacency matrix, correlation between each gene and the module eigengene (module membership), and correlation among pairwise gene expression correlations. The significance of the observed preservation statistics was determined using 200 permutation tests where gene labels are randomly assigned in the test network to estimate the mean and standard deviation under the null hypothesis of no preservation. The resulting Z-scores provide a measure of the whether the observed gene connectivity pattern of the test network is significantly more conserved than random. The median of the four Connectivity Z-scores and the median of the four density Z-scores are averaged to generate a summary Z-score [69].

#### Differential gene expression analysis

Differential gene expression across the three birth weight categories was assessed using the limma R package [62]. Briefly, limma implements an empirical Bayes method to generate gene-wise moderated t-statistics across contrasts of interests. Observed associations were considered significant at FDR < 0.05. Enrichment for differentially expressed genes in network modules was determined using Fisher’s exact test.

Figures depicting enrichment of GO terms and differentially expressed genes in network modules, differential expression of modules across birth weight categories and the forest plot of the association between hub gene expression and aberrant fetal growth were generated using the ggplot2 R package [70]. The figure highlighting differentially expressed genes and hub genes in the salmon module was generated using visANT 5.0 [71, 72]. Figures showing hierarchical clustering of network modules and network preservation measures were generated using the WGCNA R package [17]. The remaining figures were generated using R base plotting functions. All analysis was conducted using R 3.3.1 [73].

## Additional files

Additional file 1: Distribution in expression level (logCPM) among top expressed genes in placenta. Shown in grey is the distribution in expression level across all genes (*n* = 12,135). (TIFF 1487 kb)

Additional file 2: Agreement in gene expression rankings between RNAseq and NanoString (*n* = 197). Gene expression across 80 genes was assessed by both the RNAseq and NanoString platforms. Genes were ranked by expression level (lowest rank signifies highest expression across samples). Gene ranks across the platforms were significantly correlated (Spearman rho = 0.76, *p* < 0.01). (TIFF 1666 kb)

Additional file 3: Gene loadings in placental gene coexpression network. Genes loading into separate modules are listed. Additionally the correlation between genes and module eigengenes are indicated. (CSV 2815 kb)

Additional file 4: Hierarchical clustering of network modules. (TIFF 1474 kb)

Additional file 5: Mapping of GWAS-linked genes in placental gene coexpression network. Genes linked to GWAS-associated traits that are enriched in the placental gene coexpression network are listed alongside assigned modules and GWAS-linked traits. (CSV 58 kb)

Additional file 6: Candidate module hub genes relevant to birth weight. Plots indicate the correlation between gene expression and module eigengene values (x-axis) and the correlation between gene expression and birth weight category (y-axis). Genes that demonstrate both module importance and birth weight relevance are indicated for each module. Genes of interest include *MKL2*, *PSMD4*, *ADRM1*, *AC3H15* and *CREB3* in the turquoise module, *LAD1*, *KAT5*, *DNAJC14* and *BECN1* in the red module, *GRHL1*, *INHBA*, *PVRL4*, *LEP* and *C8orf58* in the salmon module, *DDX3X* in the lightcyan module, and *ZNF460*, *C21orf91* and *PAN3* in the greenyellow module. (TIFF 169 kb)
