# Supplementary figures and images for: Whole-transcriptome analysis delineates the human placenta gene network and its associations with fetal growth
Source: BMC Genomics. 2017 Jul 10;18:520. doi: 10.1186/s12864-017-3878-0 (PMC5502484; doi:10.1186/s12864-017-3878-0)

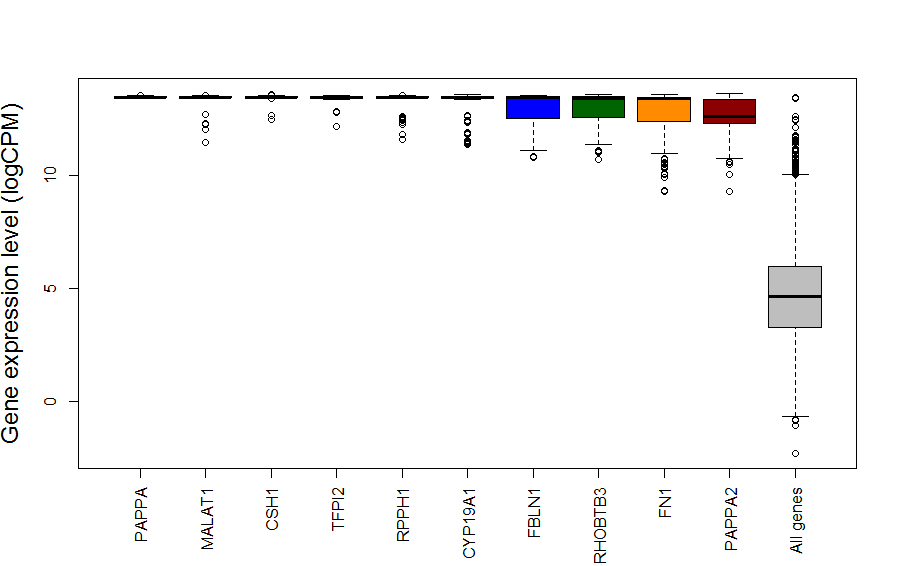

Supplement: Supplementary file 1 — Distribution in expression level (logCPM) among top expressed genes in placenta. Shown in grey is the distribution in expression level across all genes (n = 12,135). (TIFF 1487 kb) [file 12864_2017_3878_MOESM1_ESM.tiff]

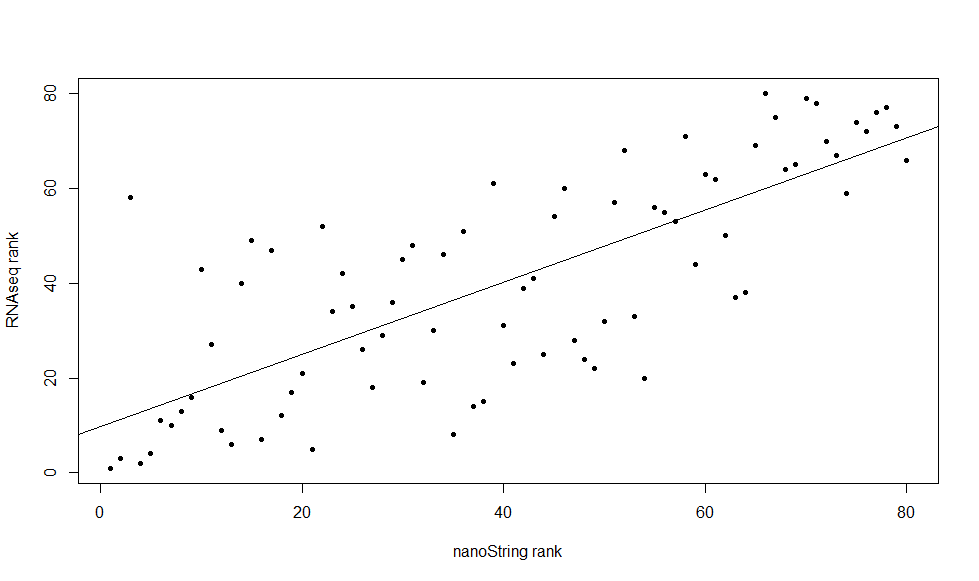

Supplement: Supplementary file 2 — Agreement in gene expression rankings between RNAseq and NanoString (n = 197). Gene expression across 80 genes was assessed by both the RNAseq and NanoString platforms. Genes were ranked by expression level (lowest rank signifies highest expression across samples). Gene ranks across the platforms were significantly correlated (Spearman rho = 0.76, p < 0.01). (TIFF 1666 kb) [file 12864_2017_3878_MOESM2_ESM.tiff]

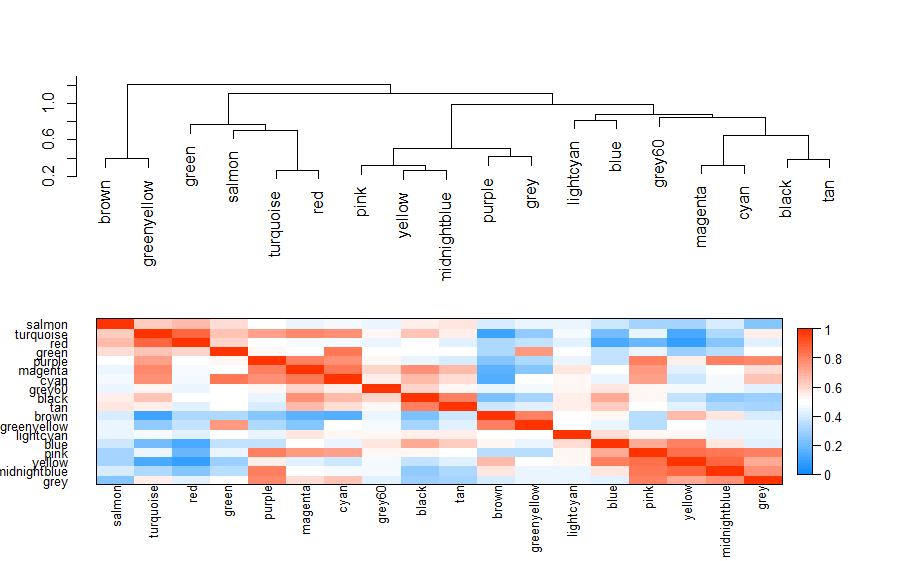

Supplement: Supplementary file 4 — Hierarchical clustering of network modules. (TIFF 1474 kb) [file 12864_2017_3878_MOESM4_ESM.tiff]

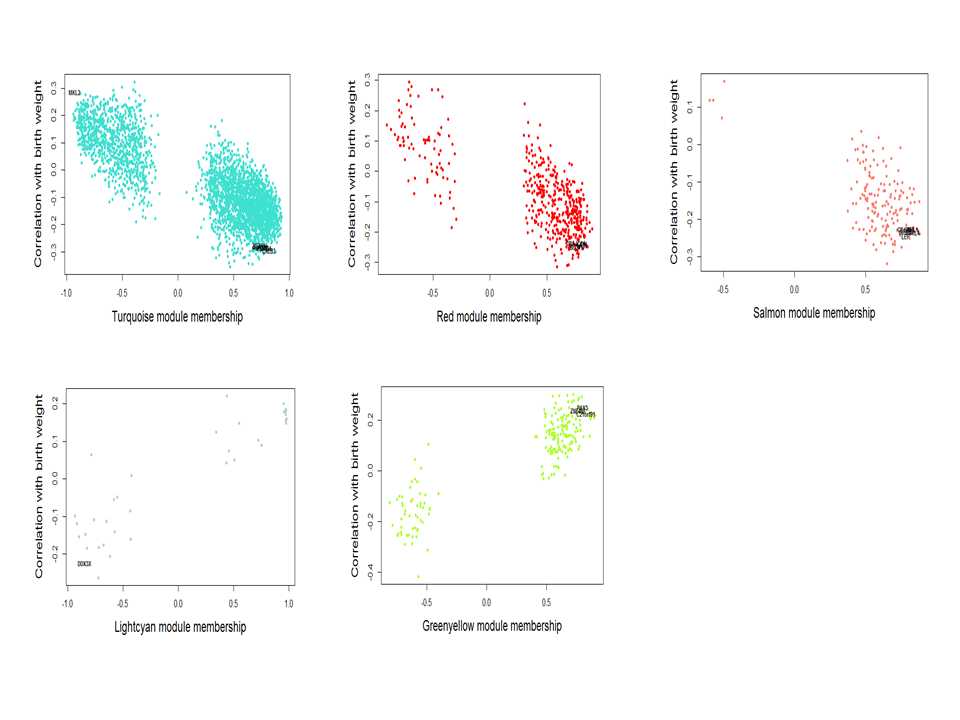

Supplement: Supplementary file 6 — Candidate module hub genes relevant to birth weight. Plots indicate the correlation between gene expression and module eigengene values (x-axis) and the correlation between gene expression and birth weight category (y-axis). Genes that demonstrate both module importance and birth weight relevance are indicated for each module. Genes of interest include MKL2, PSMD4, ADRM1, AC3H15 and CREB3 in the turquoise module, LAD1, KAT5, DNAJC14 and BECN1 in the red module, GRHL1, INHBA, PVRL4, LEP and C8orf58 in the salmon module, DDX3X in the lightcyan module, and ZNF460, C21orf91 and PAN3 in the greenyellow module. (TIFF 169 kb) [file 12864_2017_3878_MOESM6_ESM.tif]
